# Cyclic Attractors Are Critical for Macrophage Differentiation, Heterogeneity, and Plasticity

**DOI:** 10.3389/fmolb.2022.807228

**Published:** 2022-04-11

**Authors:** Manuel Azaid Ordaz-Arias, Laura Díaz-Alvarez, Joaquín Zúñiga, Mariana Esther Martinez-Sánchez, Yalbi Itzel Balderas-Martínez

**Affiliations:** ^1^ Laboratorio de Biopatología Pulmonar, Instituto Nacional de Enfermedades Respiratorias Ismael Cosío Villegas, Mexico City, Mexico; ^2^ Licenciatura en Ciencias Genómicas, UNAM, Cuernavaca, Mexico; ^3^ Departamento de Inmunología, Instituto de Investigaciones Biomédicas, UNAM, Mexico City, Mexico; ^4^ Posgrado en Ciencias Biológicas, UNAM, Mexico City, Mexico; ^5^ Instituto Nacional de Enfermedades Respiratorias Ismael Cosío Villegas, Mexico City, Mexico; ^6^ Tecnológico de Monterrey, Escuela de Medicina y Ciencias Biomédicas, Mexico City, Mexico

**Keywords:** boolean network, macrophage, oscillation, cycles, differentiation, heterogeneity, plasticity, regulatory network

## Abstract

Adaptability, heterogeneity, and plasticity are the hallmarks of macrophages. How these complex properties emerge from the molecular interactions is an open question. Thus, in this study we propose an actualized regulatory network of cytokines, signaling pathways, and transcription factors to survey the differentiation, heterogeneity, and plasticity of macrophages. The network recovers attractors, which in regulatory networks correspond to cell types, that correspond to M0, M1, M2a, M2b, M2c, M2d, M2-like, and IL-6 producing cells, including multiple cyclic attractors that are stable to perturbations. These cyclic attractors reproduce experimental observations and show that oscillations result from the structure of the network. We also study the effect of the environment in the differentiation and plasticity of macrophages, showing that the observed heterogeneity in macrophage populations is a result of the regulatory network and its interaction with the micro-environment. The macrophage regulatory network gives a mechanistic explanation to the heterogeneity and plasticity of macrophages seen *in vivo* and *in vitro*, and offers insights into the mechanism that allows the immune system to react to a complex dynamic environment.

## Introduction

The balance between inflammatory and anti-inflammatory immune responses is crucial to maintain homeostasis in the face of the diverse immune challenges an organism meets. Macrophages are cells essential to immunity. They recognize pathogens and pathogen-derived molecules, collaborate with other cells of the innate and adaptive immune system, and are critical players both in chronic inflammation and in tissue regeneration (Sica and Mantovani, 2012; Varol et al., 2015; Vannella and Wynn, 2017; Li et al., 2019; Locati et al., 2020). Macrophages are characterized by their diversity and plasticity. Depending on the signals received, non-polarized M0 macrophages can be polarized into two main types: classically activated macrophages or M1, characterized by a pro-inflammatory profile, and alternatively activated macrophages or M2, which promote proliferation and repair (Mendoza-Coronel and Ortega, 2017; Funes et al., 2018).

M0 macrophages are usually monocytes differentiated into M0 macrophages in the presence of GM-CSF that have not been exposed to any pro or anti-inflammatory stimulus or environment that promotes their activation, cytokine production, and functional polarization (Kumar, 2019). M1 polarization is generally triggered by the stimulation of TLRs, or by cytokines such as IFNγ and GM-CSF, which lead to high production of pro-inflammatory cytokines such as IL-1β, IL-6, IL-12, and IL-23, in addition to a low expression of IL-10 (Lehtonen et al., 2002; Park et al., 2009; Weber et al., 2010; At et al., 2011; Lawrence and Natoli, 2011; Liu et al., 2014; Bally et al., 2015; Funes et al., 2018; Hamilton, 2019; Wang et al., 2019; Petrina et al., 2021). M2 polarization has been subdivided into M2a, M2b, M2c, and M2d macrophages, due to diverse transcriptional programs and stimuli involved (Huang et al., 2018). The M2a macrophages are derived from M0 cells stimulated by IL-4 and IL-13, they release high levels of IL-10 and TGF-β using transcriptional factors (TFs) such as STAT6 and IRF4, and are involved in proliferation and tissue repair functions (Bouhlel et al., 2007; Chawla, 2010; Gordon and Martinez, 2010; Ma et al., 2015; Arora et al., 2018; Wang et al., 2019). A combination of TRL ligands generates M2b macrophages and immune complexes (IC) as well as IL-1R ligands, yielding both pro-inflammatory cytokines, such as IL-1β, IL-6, TNF-α, and IL-12, and anti-inflammatory cytokines such as IL-10. The signaling pathways stimulated involve MAPKs, PI3K/Akt, and, ultimately, NF-κB. M2b macrophages have a role in the regulation of inflammatory responses (Lucas et al., 2005; Park et al., 2009; Zhang et al., 2009; Luo et al., 2010; Weber et al., 2010; Foey, 2014; Liu et al., 2014; Bally et al., 2015; Wang et al., 2019). M2c macrophages arise upon IL-10 stimulation. They express high levels of IL-10, which induces the phosphorylation of STAT3, thus negatively regulating the production of pro-inflammatory cytokines (Hutchins et al., 2013; Ma et al., 2015; Wang et al., 2019). M2d macrophages are induced by the costimulation of the adenosine A2 receptor and TLR, expressing levels of IL-10 as well as IL-12, and characterized by presenting properties of tumor-associated macrophages that carry out angiogenesis and tumor progression (Leibovich et al., 2002; Grinberg et al., 2009; Park et al., 2009; Colin et al., 2014; Arora et al., 2018; Wang et al., 2019; Anders et al., 2021). However, these are not the only possible expression patterns, as variations have been found *in vitro* and *in vivo*.

Given th e plasticity, heterogeneity, and adaptability of macrophages and their role in the immune system, it is important to understand their phenotypic landscape, the conditions in which they originate, and the possible transitions between subsets. Macrophage differentiation can be seen as a continuum between M1 and M2 phenotypes, where these cells can express different profiles and concentrations of cytokines, receptors, and transcription factors (Sica and Mantovani, 2012; Palma et al., 2018). At the same time, not all combinations of key molecules like IL-12, IL-10, IL-6, or VEGF are possible. For example, the IFNγ-induced and IL-4-induced programs inhibit each other in the cell, leading to heterogeneous populations in environments with mixed signals (Munoz-Rojas et al., 2021). Furthermore, it is known that the signaling pathways have inhibitory mechanisms that lead to self-regulation, causing oscillations in the expression of cytokines like IL-6, which are expected as part of the physiological behavior of macrophages, but that can also act against the host in pathological scenarios (Wang et al., 2013).

Regulatory networks are a valuable tool to bridge the molecular regulation of a cell with its phenotype and have been used to study the differentiation of hematopoietic cells (Saez-Rodriguez et al., 2007; Naldi et al., 2010; Martinez-Sanchez et al., 2015; Liquitaya-Montiel and Mendoza, 2018; Ramırez and Mendoza, 2018; Palma et al., 2018; Ramirez et al., 2019; Avila-Ponce de León et al., 2021) and the plasticity of macrophages (Palma et al., 2018; Ramirez et al., 2019; Avila-Ponce de León et al., 2021). Boolean networks integrate qualitative data about the interactions between cytokines, signaling pathways, and transcription factors to predict differentiation and plasticity. They allow testing different hypotheses and determine how the regulatory structure impacts complex cellular behaviors, all of this, with only a few parameters (Kauffman, 1969; Albert and Thakar, 2014).

This paper presents a Boolean model of the regulatory network that underlies macrophage differentiation, extending previous approaches (Palma et al., 2018; Ramirez et al., 2019). The model recovers the M0, M1, M2a, M2b, M2c, M2d, and other M2-like cell types, including several cyclic attractors that reproduce known experimental data. Then, we use the model to study how the classic polarizing environments and mixed combinations of extrinsic signals affect the stability of these cells. We show that the plasticity, heterogeneity, adaptability, and variable levels of expression of key cytokines in macrophages result from the structure of the regulatory network.

## Materials and Methods

All the datasets, scripts, tables, and images used in this study can be found in the repository https://github.com/mar-esther23/Macrophage_Differentiation.

A Boolean network consists of nodes representing molecular components (i.e., cytokines, signaling pathways, transcription factors) and edges representing the interactions between them. The value of the nodes is a discrete variable: one if the node is functional and 0 if it is not functional. The value of a node i at the time *t+1* depends on the value of its regulators at time *t*, according to a logical function that recapitulates available biological information. The state of the network at *x(t)* depends on the values of all its nodes and will evolve through time as the regulatory functions are evaluated. Eventually, the system will arrive at an attractor, which corresponds to a cell type. These attractors can be steady states when x_t_ = x_t+1_ or cycles when x_t_ = x_t+τ_ (Kauffman, 1969; Albert and Thakar, 2014).

**FIGURE 1:**
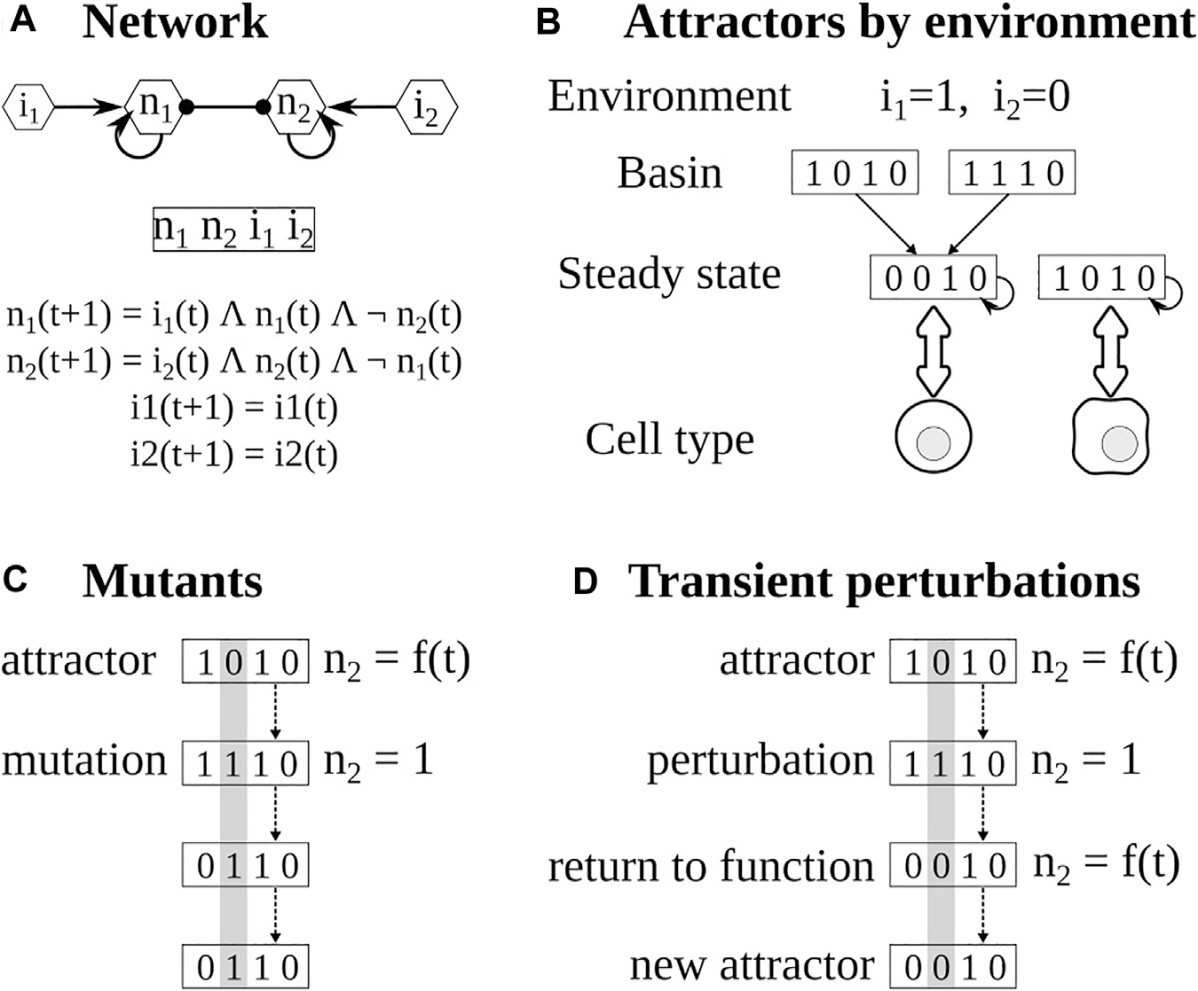
Pipeline for the analysis of Boolean networks. **(A)** The network is constructed using available experimental information. The state of the network depends on the values of each node. The value of each node depends on its regulators. **(B)** The attractors of the network are calculated using BoolNet, the attractors depend on the inputs or environment and the functions. **(C)** Mutants are obtained by fixing the value of the target node for the whole simulation. **(D)** In transient perturbations, the target node is changed (bitflip) for one time step, and then the perturbation is relaxed. Eventually, the network may stay, return to the original attractor, or reach a different one.

We constructed the macrophage regulatory network according to previous models (Palma et al., 2018) and available information (Leibovich et al., 2002; Yoshimura et al., 2007; Chang et al., 2013; Wang et al., 2013; Liu et al., 2014; Wilson, 2014) among others which can be seen in (Supplementary Table S1, Figure 1A). The dynamical analysis of the network was done using the packages BoolNet (Mussel et al., 2010) and BoolNetPerturb (Martinez-Sanchez et al., 2018). Synchronous updating was used in all simulations.

We determined attractors of the network and classified them depending on the expression of both the characteristic transcription factor and cytokine. In every case, we focused on the presence and absence of the nodes that correspond to common cell type markers, and ignored the value of the other nodes (Kauffman, 1969; Albert and Thakar, 2014; Martinez-Sanchez et al., 2015). The basin of attraction of a network is the set of states that lead to an attractor (steady state or cycle) in the simulation.

To determine the effect of the micro-environment, we first determined the cytokines present in each polarizing environment (Kauffman, 1969; Albert and Thakar, 2014; Martinez-Sanchez et al., 2015), then, we fixed the corresponding input nodes according to the cytokines present or absent in that environment and then determined the resulting attractors (Figure 1B). This is modeled by changing the input function i_t+1_ = i_t_ to i_t+1_ = = 0 or i_t+1_ = 1 depending on the presence or absence of the cytokines in the environment.

To further verify the model, we simulated the knock-out and overexpression of target nodes by setting their values to 0 or 1 and comparing the resulting attractors with known mutants (Kauffman, 1969; Albert and Thakar, 2014; Martinez-Sanchez et al., 2015) (Figure 1C). Furthermore, we checked that the attractors found with synchronous updating could be found using asynchronous updating.

The expression pattern of a cell can change in response to changes in both internal and environmental factors. We focused on the effect of small transient perturbations. For example, due to stochastic effects, a transcription factor may not be activated because the polymerase fails to bind to its DNA sequence for a time, even if the rest of the regulators are present. This can be modeled as the corresponding node having a value of zero for a time step, and then the perturbation will be relaxed and the node will acquire a new value depending on its regulators (Figure 1D). On the other hand, a cell may be subjected to a small peak of a cytokine in its environment. This can be modeled as the extrinsic cytokine node having a value of one for a time step and then returning to its original value. The attractor of the system, which corresponds to the cell type, may change or not depending on the regulatory network, the original state of the network, and the perturbed node. To study the stability and plasticity of the system for each microenvironment we took its attractors and modified for one time step the value of the node (bitflip), then the perturbation was relaxed, and the resulting attractor was determined (Martinez-Sanchez et al., 2018, 2015).

## Results

### Macrophage Differentiation Patterns Emerge From Feedback Between Transcription Factors, Cytokines, and Signaling Pathways

**FIGURE 2:**
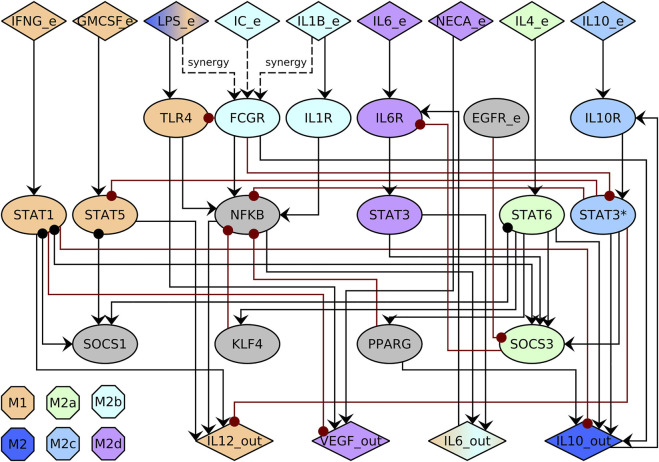
Macrophage regulatory network. The network includes cytokines in the environment (_e) and produced by the macrophage (_out), signaling pathways, and transcription factors (ellipses). Activations are represented with black arrows, and inhibitions with red dotted arrows. We use STAT3 for IL-6 dependent signaling and STAT3* for IL-10 dependent signaling. The color of the node corresponds to the associated cell type.

We expanded the previously published macrophage regulatory networks (Palma et al., 2018; Ramirez et al., 2019). In this network, we included multiple molecules like transcription factors, STAT proteins, cytokine receptors, SOCS proteins, and cytokines, among others. We only included direct interactions that have been experimentally validated (Supplementary Table S1, Supplementary Table S2) to include Ie IL6 (Chang et al., 2013; Wang et al., 2013; Liu et al., 2014; Wilson, 2014), NECA (Leibovich et al., 2002), EGFR (Wang et al., 2013), and SOCS3 (Yoshimura et al., 2007; Wang et al., 2013; Wilson, 2014). Then, we simplified the network using GINSIM(Gonzalez et al., 2006). The resulting network has 29 nodes and 52 interactions (Figure 2, Supplementary Table S3). We assumed that different pathways mediate IL-6 and IL-10 signaling by STAT3 and marked them as STAT3 for IL-6 dependent signaling and STAT3* for IL-10 dependent signaling. The state of a node represents whether the biological component is active 1) or inactive (0). A node is active if it can alter the regulation of other nodes.

**FIGURE 3:**
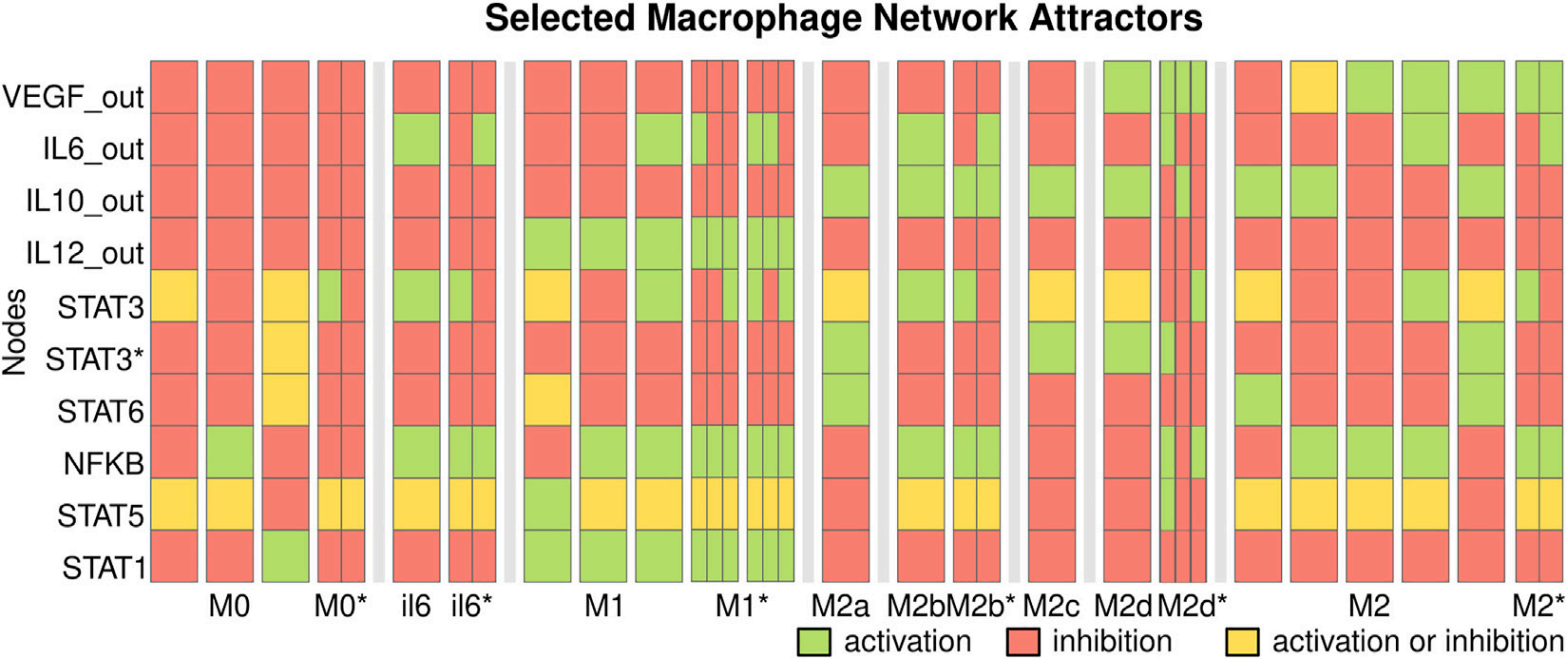
Selected macrophage regulatory attractors. The attractors of the macrophage regulatory network correspond to cell types. Each column corresponds to key nodes of a state; attractors are separated by white spaces and cell types by black bars. We include the cyclic attractors as narrow columns that represent the oscillation. Each node can be active (green) or inactive (red), or active or inactive (yellow). The network recovers the attractors corresponding to M1, M2a, M2b, M2c, M2d, and M2-like cell types.

Then, we determine the macrophage cell types by calculating the attractors, steady states, and cycles of the network (Kauffman, 1969) and label them. An attractor corresponds to a cell type if the characteristic signaling pathways, transcription factors, and produced cytokines are present (Supplementary Table S4). The network recovers 44 steady-state attractors and 358 cyclic attractors of size 2, 3, and 6 which correspond to ‘M1’, ‘M2b’, ‘M2a’, ‘M2c’, ‘M2d’, ‘M2’ (M2-like), ‘M0’, and ‘il6’ cell types (Figure 3, Supplementary Figure S2, Supplementary Table S5).

M1 macrophages produce IL-12 and may produce IL-6 and activate the pathways for STAT1, STAT5, or NFKB. M0 and cyclic M0* macrophages do not produce any cytokines and correspond to naive macrophages. They are usually found in simulated environments with neither extrinsic cytokines nor contradictory extrinsic signals that inhibit each other through SOCS proteins. The attractors labeled ‘il6’ produce IL-6 but no other extrinsic cytokines. The steady states il6 macrophages are only found when there is EGFR_e in the microenvironment and may correspond to inflammatory pathogenic states as those seen in cancer (Wang et al., 2013) and severe COVID-19 (Matsuyama et al., 2020; Merad and Martin, 2020). In contrast, the cyclic il6* attractors may correspond to pathogenic states or be non-fully differentiated macrophages.

M2 macrophages produce IL-10 or VEGF, and they can be classified into different subtypes depending on the cytokines produced and active signaling pathways. The M2a subtype produces IL-10 and activates the STAT6 pathway, M2b produces IL-10 and IL-6, M2c produces IL-10 and activates the IL-10 dependent STAT3* pathway, M2d macrophages produce VEGF, IL-10 and no IL-12. The model recovers steady states corresponding to these cell types, including cyclic attractors for M2b with an IL-6 dependent STAT3 oscillation. We also recover M2-like subsets that produce IL-10 and VEGF or IL-6.

Most cyclic attractors present oscillations in the IL6/STAT3/SOCS3 circuit, which may affect the downstream production of IL6_out. The self-inhibition of the IL6 pathway causes these oscillations: STAT3 induces SOCS3 expression, which inhibits IL6R and STAT3 phosphorylation, causing a repressed circuit and oscillations. The IL10/STAT3* pathway also presents oscillations that may affect SOCS1, NFKB, and STAT5. These oscillations can be caused by inhibition by other pathways, for example, IL6. The self-inhibition caused by SOCS3 and the other cycles may have a role in limiting the production of IL-6 by macrophages and the associated hyperinflammation. Given the hypothesis that macrophage differentiation is a continuum (Sica and Mantovani, 2012; Palma et al., 2018) these M2-like and cyclic states may be a mechanism to regulate the production of cytokines by macrophages.

The number of attractors associated with a cell type does not necessarily correspond to the number of states that reach the attractors of that cell type (basin of attraction). The biggest basin is M0, followed by M2, M2a, and M1, while il6, M0*, and il6* had the smaller basins. In general, the cell types labeled as cyclic (*) had smaller basins.

To validate the model we verified whether the attractors were robust to asynchronous updating. All the steady states were robust to the change in update schema. While most of the attractors of sizes 2 and 3 were unstable we found asynchronous attractors of size 6 or more that correspond to M0*, il6*, M2b*, and M2*, but lost the M1* and M2d*.

To further validate the model, we compared the knock-out and over-expression simulations with experimental data (Supplementary Figure S3, Supplementary Table S6). In general, the predictions made by the model correspond to the observed biological data (Supplementary Table S7). For instance, in STAT1-null macrophages stimulated with IFN-γ and Pam3CSK4, a dose-dependent decrease in IL-12 has been experimentally observed compared to wild-type macrophages (Kim et al., 2015). This phenomenon is recovered by a network simulation of a STAT1 knock-out, where we see that attractors completely lose IL-12 production, causing the disappearance of M1. In the same way, it has been experimentally obtained that inhibition of PPARγ-dependent gene expression significantly decreases the production of IL-10 mediated by LPS, which is recovered in the simulations in which a knock-out of PPARγ was set, obtaining a decrease in IL-10-producing attractors (Majai et al., 2007). Furthermore, regarding overexpression, there is the experimental case where *in vitro* STAT6 has been overexpressed, causing a promotion of M2 macrophages (Gong et al., 2017). An overexpression simulation in STAT6 recovers this last, which causes a higher proportion of M2 attractors and a decrease in M1. However, these simulations do not recover the expected behavior in the case of the NFkB mutant. The NF-kB pathway is a highly complex protein, but our network simplifies it to a single node, so this discrepancy is probably the result of the modeling decisions. In this mutations experiments, the most stable states were the M0*, il6*, and M2d macrophages. On the other hand, the more sensitive cell types are M0 and M1. The nodes that tended to cause more changes in differentiation if mutated are IL12_out and IL10_out, which affect the cytokine profile, followed by STAT1, STAT6, and IL-10 mediated STAT3 activation (STAT3*). Furthermore, this analysis predicts the effect of knock-out and over-expression mutants that have not been tried experimentally.

### Role of the Micro-Environment in Macrophage Differentiation and Stability

**FIGURE 4:**
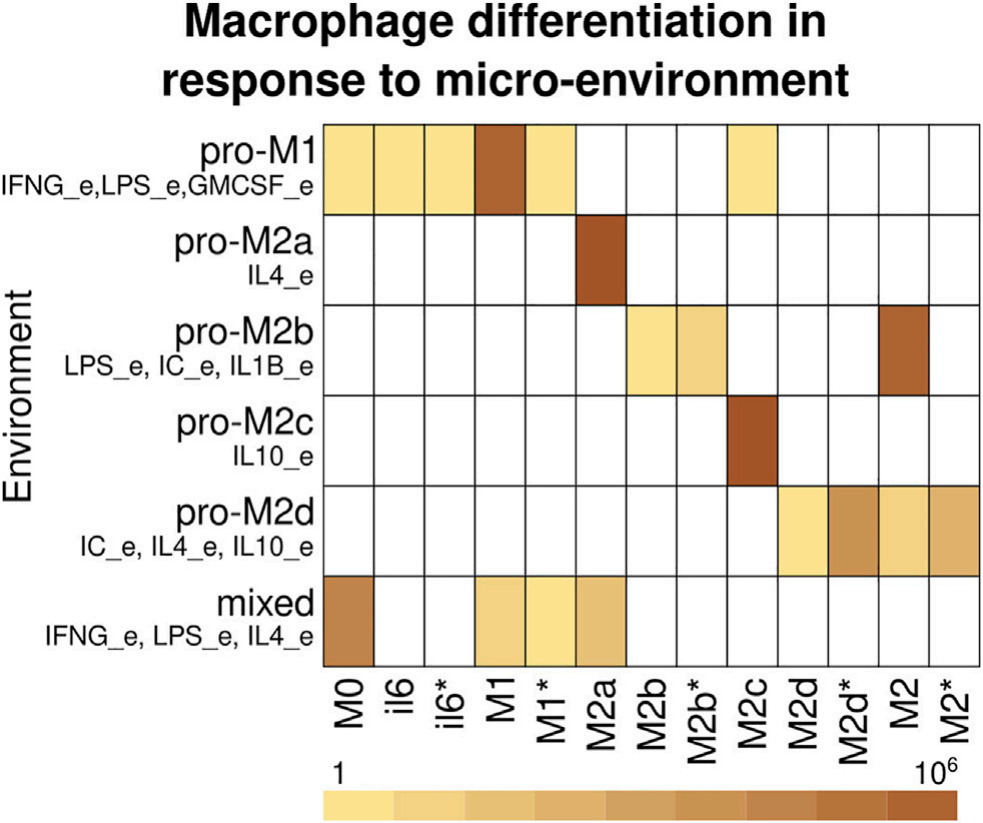
Macrophage differentiation in response to the micro-environment. Cell types recover depending on the cytokines in the micro-environment. The rows correspond to the environment and the columns to the cell type. The color corresponds to the basin size of the attractors that correspond to each cell type. If a cell type was not recovered on a microenvironment, it is represented with white.

Macrophage differentiation does not occur in a vacuum but in response to the micro-environmental signals (Supplementary Table S8). To determine the role of the microenvironment, we determined the attractors associated with a cell type and their combined basin size in different microenvironments (Figure 4, Supplementary Table S9). The pro-M1 microenvironment contains IFNG_e, GMCSF_e, and LPS_e, and the model mainly recovers the presence of M1 attractors with a small number of M1*, M0, il6, il6* attractors. The pro-M2a environment contains IL4_e, and the model recovers only M2a attractors. The pro-M2b environment contains LPS_e, IC_e, and IL1B_e, and the model recovers M2, M2b, and M2b*. The pro-M2c environment contains IL10_e, and the model recovers only M2c attractors. The pro-M2d environment contains IC_e, IL4_e, and IL10_e, and the model recovers M2d, M2d*, M2, and M2* attractors. The mixed environment contains LPS_e, IFNG_e, and IL4_e, which are associated with M1 and M2a polarization, and recovers M0, M1, M1*, and M2a attractors, which are in accordance with the heterogeneity observed in macrophage populations subjected to *in vitro* co-stimulation with these same cues (Munoz-Rojas et al., 2021).

**FIGURE 5:**
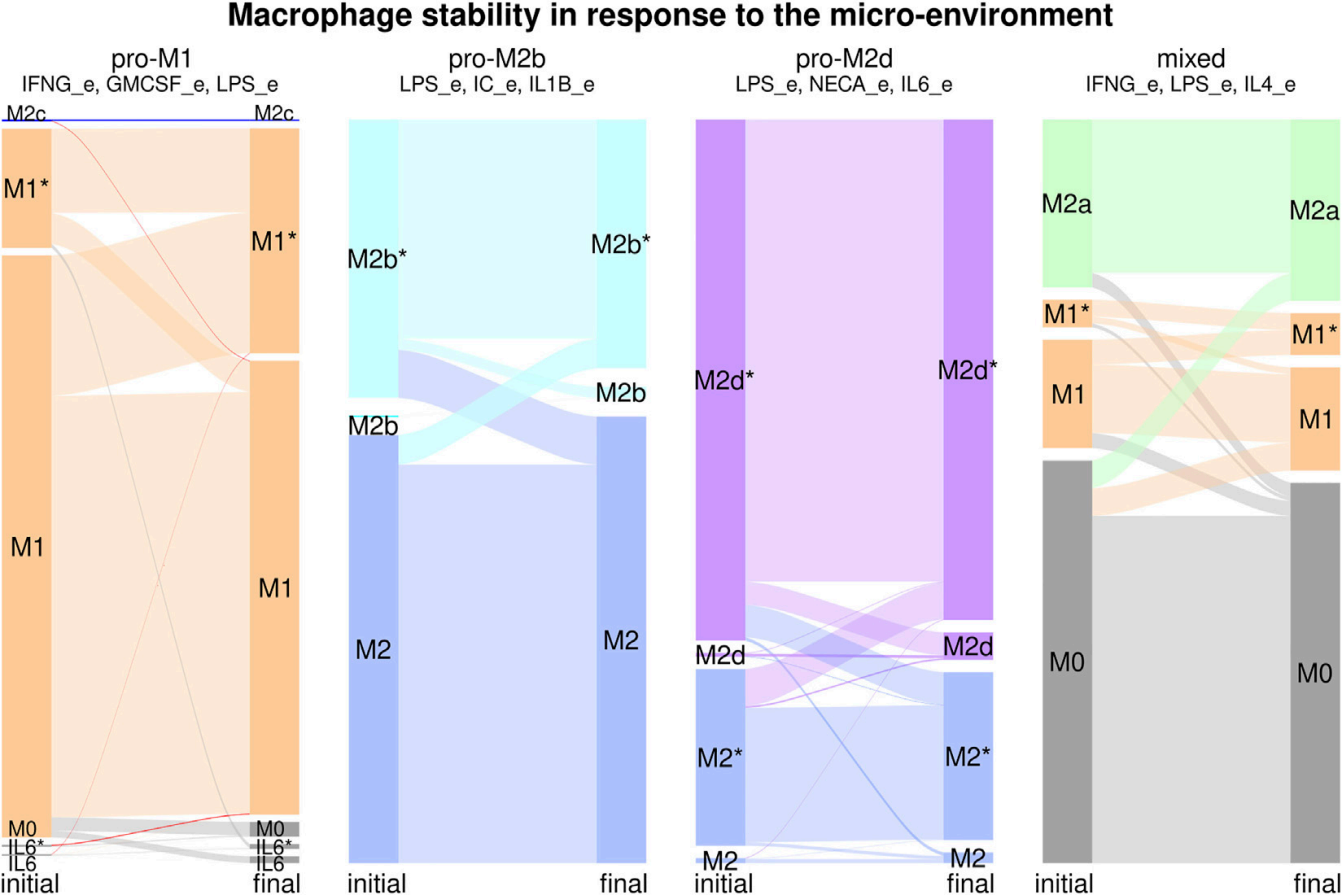
Macrophage stability in response to the microenvironment. For each environment we calculated the attractors, then, for each attractor, we transiently perturbed every node independently for one time step to determine the stability of the different cell types. Each stability experiment is represented by a flux diagram, where the colored boxes correspond to each cell type. The initial state is on the left of the diagram, and the final state is on the right. The height of the bar corresponds to the basin size of the attractor. The width of the lines between boxes represents the transitions between attractors.

Macrophage differentiation is not a wholly deterministic process, but it can be affected by transient changes in the environment, stochastic noise during transcription, traduction and signaling events, and other types of noise. Furthermore, the environment and the internal state of the cell can have small changes in response to the progress of a pathological state. To study this for each of the six environments, we took the recovered attractors and perturbed each node one by a time step, and determined if that changed the resulting attractor and cell type (Figure 5, Supplementary Table S10). In the pro-M1 environment, most of the transitions are between M1 and M1*, with a bias towards the cyclic M1* attractors. Most M1* attractors have oscillations in the IL6/STAT3/SOCS3 pathway, and some of them have oscillations in IL6_out. There is a small number of transitions towards M0 and il6/il6* that increase in percentage, which may have a role *in vivo* by limiting the production of IL-12 as the infection progresses toward resolution. In the pro-M2b environment, there is a small number of transitions between M2 and M2b/M2b*, with a slight bias towards M2. In the pro-M2d environment, there are also transitions between M2/M2* and M2d/M2d*. In the pro-M2a and pro-M2c environments, there are only M2a and M2c attractors, so these are stable. In the mixed environment, there are transitions from M0 to and from all differentiated environments but not between M1 and M2a attractors, which may indicate that the plasticity between these cell types requires longer signals, especially in mixed environments, and that a temporal cease of cytokine production precedes a transition between M1 and M2 cell types.

In general, we can say that each microenvironment favors the differentiation and stability of the associated cell type, even in some cases where there is a small number of attractors associated with an additio’nal cell type. The exceptions are the pro-M2b and pro-M2d environments, where there is a strong presence of M2-like attractors; however, this can be seen as part of the phenotypic plasticity of macrophages. If we consider all possible combinations of cytokines, most of the cell types were highly stable, with only a small proportion of transitions between subsets. The cyclic attractors associated with a cell type were highly stable, as the oscillations seem to be the result of the network topology and not a dynamical artifact.

**FIGURE 6:**
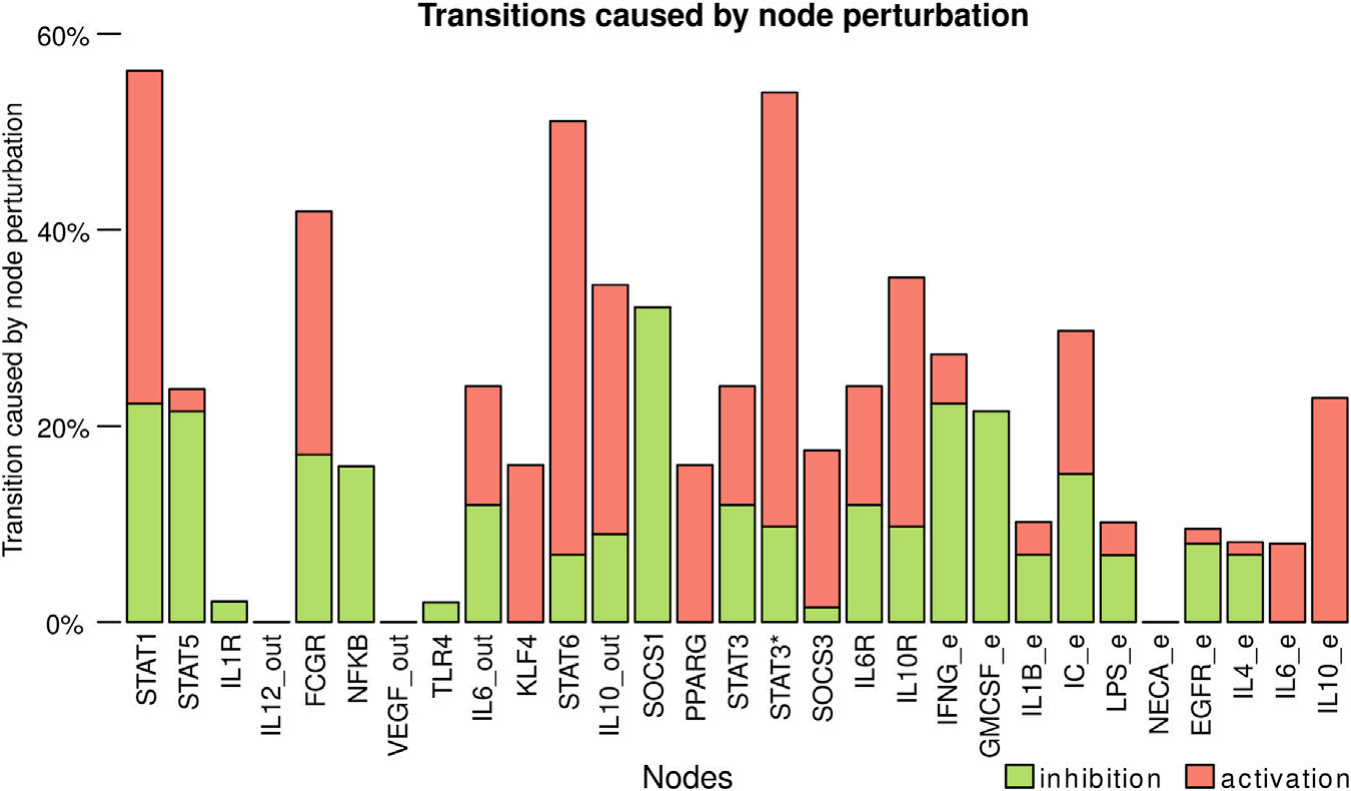
Transitions caused by node perturbation. Each bar corresponds to the percentage of perturbations of a node that caused a transition between cell types. Activation of the nodes is represented with green and inhibitions with red. https://github.com/mar-esther23/Macrophage_Differentiation/blob/master/images/MP_transitionnode_clean.png.

To determine the key nodes for the dynamic stability of the model, we determined which nodes caused more changes between cell types when transiently perturbed (Figure 6, Supplementary Table S10). The nodes that caused more transitions between cell types were STAT1, IL-10 mediated STAT3*, and STAT6, which are associated with the signaling pathways of key cytokines in macrophage differentiation. IC_e and FCGR also had an essential role in the stability of the model, as they regulate both NFKB, STAT3*, and IL10_out. STAT1, STAT3*, STAT6, and FCGR have a higher number of out-going edges and directly or indirectly modulate the activation and inhibition of different circuits of the network. The activation of SOCS1 also has a relevant role, as its activation inhibits the STAT1, STAT5, and STAT6 nodes. The nodes that cause fewer transitions between cell types are IL12_out, VEGF_out, NECA_e, IL1R, and TLR4.

In general, of the single state transient perturbations 21% resulted in a change of cell type and 2.96% were transitions between M1 and M2 states. We also simulated all possible double node perturbations 34.44% resulted in a change of cell type and 5.02% were transitions between M1 and M2 states. We also realized a Derrida curve (Derrida and Pomeau, 1986) to determine how sensible the system was to perturbations in the value of the states. For perturbations of Hamming distance 1 on average the change was of 4.18 nodes and increased towards 8.47 nodes on average as the number of perturbed nodes increased (Supplementary Figure S3). It is worth taking into account that the high number of input nodes, that represent the micro environment, heavily influence these results. For example, on a micro-environment with only M2 attractors it is impossible for there to be a transition towards M1, as the cell type is not stable. Furthermore, not all possible micro-environments can be found *in vivo*, which implies that while a transition may be possible in the system it might not be observed *in vivo*.

## Discussion

In this paper, we propose an actualized regulatory network of cytokines, signaling pathways, and transcription factors to study the differentiation, heterogeneity, and plasticity of macrophages. This network allows us to give a mechanistic explanation of the dynamic behavior of these cells in response to different micro-environments. Furthermore, the network recovers multiple cyclic attractors that are both stable to perturbations and in accordance with previous experimental observations (Wang et al., 2013), showing that these oscillations are the result of the structure of the network and suggesting that they may have a biological function. In fact, the biological relevance of oscillatory behavior seems to lie in exquisitely regulated phenomena like the activation and nuclear translocation of NFKB. Cheng et al. (2021) recently demonstrated that macrophages respond to different proinflammatory micro-environments with oscillatory or non-oscillatory activity of NFKB. ChIP-seq data and computational modeling analysis reveal that NFKB presents oscillatory behavior in the majority of stimulated cells, however, only non-oscillatory behavior leads to sufficiently prolonged chromatin accessibility, favoring gene expression. This phenomenon is regulated by the NFKB inhibitor IkBa, which may allow sensing of the micro-environment while refraining the cell from secreting inflammatory mediators until the concentration of a certain cue reaches a specific threshold. All of it without changing the cell phenotype. Therefore, we can also speculate that inflammatory disorders may arise or be sustained by macrophages whose oscillation is skewed towards non-oscillatory behavior. The macrophage regulatory network recovers steady state and cyclic attractors that correspond to M0, M1, M2b, M2d, M2-like, and IL-6 producing macrophages. Cyclic attractors represent 89% of the total attractors, but their combined basins of attraction are only 11% of the total state space. However, it is worth noting that most of these attractors are stable, and when perturbed, most perturbations lead to a cycle of the same cell type. Oscillatory activation of STAT3 with its downstream effect in IL-6 production in macrophages has been previously reported (Wang et al., 2013). The oscillations in the macrophage regulatory network are the result of the IL-6R/STAT3/SOCS3 pathway, and the crosstalk with other signaling pathways, like those mediating the activation of STAT3* via STAT6, STAT5, or IL-10, which are circuits commonly observed in immune cell regulatory networks.

*In vivo* and *in vitro* macrophages express marker molecules and cytokines in an expression range, which can be observed in a flow cytometric analysis as the spread of the population on a dot plot or the width of a histogram (Munoz-Rojas et al., 2021). The expression range can vary depending on the cell type, the molecule being measured, and the pathological state. For example, the levels of IL-6 produced by macrophages in cancer and COVID-19 are associated with the severity of the disease (Wang et al., 2013; Matsuyama et al., 2020; Merad and Martin, 2020). How do macrophages, and other immune cells, generate and regulate the expression range is an open question. Oscillations, and their associated cyclic attractors, could be a mechanism to create variability in the expression range of a molecule. For example, when averaged over time, the oscillations in STAT3 activation, and their downstream targets in M1 macrophages could create diverse expression levels that depend on the structure of the network. These oscillatory circuits could be further finely tuned by other mechanisms like the induction of signaling pathways, transcriptional regulation or stochastic effects like variations in local cytokine concentration, noise in signaling pathways, transcription factors binding, etc. Cyclic attractors are usually ignored when studying Boolean dynamics in hematopoiesis (Alvarez-Buylla Roces et al., 2018), but the relevance of the oscillatory dynamics *in vivo* and in this network (Wang et al., 2013; Munoz-Rojas et al., 2021) indicates that more methods should be developed to study cyclic attractors and determine when and how they have a functional role.

The macrophage regulatory network also allows us to study the effect of the environment on the heterogeneity and plasticity of macrophages. All the polarizing environments favor the attractor associated with it, for example, in a pro-M2a environment, we found M2a attractors. At the same time, in most of the environments we studied (pro-MI, pro-M2b, pro-M2d, and mixed), there was more than one possible cell type, which implies that the heterogeneity in macrophages populations is a result of the regulatory network. This was especially notable in the mixed environment (LPS + IFNγ + IL4), where we recover M0, M1, and M2a macrophages (Munoz-Rojas et al., 2021). The specific differentiation pathway a cell follows is also a result of the regulatory network, the internal state of the cell, and stochastic events. Studying the basins of attraction of the different cell types and their sensibility to stochastic events may allow us to understand the heterogeneity of macrophage populations better. For example, *in vitro* stimulation with a combination of LPS, IFN-γ and IL-4 produces heterogeneous populations with M1 and M2 sub-populations (Munoz-Rojas et al., 2021). In this study Muñoz-Rojas et al. use a combination of molecular and cell biology techniques, including single-cell RNA sequencing (scRNA-seq), to ascertain the global transcriptional programs that lead to the observed heterogeneity. Similar results have been observed *in vivo*, where scRNA-seq of macrophage populations has also shown the coexistence of two clearly defined subpopulations in adipose tissue that do not follow the classic M1/M2 paradigm and whose proportions vary depending on the micro-environment (Grosjean et al., 2021). The differentiation of each individual cell depends on the initial state of the cell (transcription factors expressed and active signaling pathways) and stochastic events (local cytokine concentration, noise in signaling pathways, transcription factors binding, etc.), which generates an initial variability. Such variability determines which pathways of the regulatory network activate and which to inhibit, to polarize the individual cells into clearly defined subpopulations, thus maintaining a heterogeneous population. These results coincide with our findings that in mixed environments M0, M1, and M2 macrophages coexist, implying that the design and performance of our network are appropriate to recover the outcomes of complex scenarios reported *in vivo* and *in vitro* after extensive analyses.

The model also allowed us to study macrophage plasticity. The environment determines plasticity because it limits the accessible cell types and modulates the effect of perturbations. In general, most perturbations did not cause changes in the labeled cell type. However, transitions between a steady state and a cyclic attractor of the same cell type were common, which means that cells that may be classified as the same cell type given their membrane markers may have different internal states, creating a hidden source of heterogeneity to respond to changes in the micro-environment. In polarizing environments (pro-M1 and pro-M2), most of the transitions were towards the favored cell type. There was a high level of transitions among the different subtypes of M2 attractors but limited transitions towards M1. This seems to indicate that M2 attractors are more closely related to one another than to M1 attractors, fine tuning their regulatory activity and creating a continuum of M2-like states. The multiple inhibitions between M1 and M2 transcriptional programs help the system maintain a stable inflammatory or regulatory program, making these the two poles of macrophage differentiation. This is especially relevant when taking into account the key role of macrophage differentiation and plasticity in COVID-19 and cancer (Wang et al., 2013; Li et al., 2019; Matsuyama et al., 2020; Merad and Martin, 2020). Perturbations that favor M2 macrophages can favor transitions towards more aggressive cancers, even in situations where perturbing the cancer cells may not be enough to change the steady state behavior of the system (Li et al., 2019). This seems to imply that there are a series of feedback loops between the tissues, the environment, and immune cell populations that are crucial to understanding complex diseases. Understanding these feedback loops will require us to conceive disease as a system where the cytokine and cellular environment play a key role.

The mixed environment (LPS + IFN-γ + IL4) had a high number of non-differentiated M0 attractors, because the mixed signals most likely inhibited each other, as reported by Munoz-Rojas et al. (2021), where LPS + IFN-γ, and IL-4 give rise to orthogonal global transcriptional programs We observed only M0-M1 and M0-M2a transitions but no M1-M2a direct transitions. M1-M2a transitions are possible if they pass through an intermediate M0-like state with no cytokine production and require more than one perturbation. Such a phenomenon was indeed described by Tarique et al. (2015) for LPS + IFN-γ (M1), and IL-4 + IL-13 (M2) polarized macrophages, where the depletion of cytokines in the culture medium causes the cells to revert to the M0 phenotype, and by supplying the appropriate stimuli the macrophages can be re-polarized to the alternative phenotype. This could be a mechanism to warrant stability in the different cell types while allowing for plasticity if the environment changes past a certain time threshold. It also shows once more the power of experimental recapitulation of our network.

Traditionally, differentiation of hematopoietic cells has been considered a hierarchical process with clear differentiation pathways and well-defined cellular types, for example, M1 and M2 macrophages. However, as our understanding of macrophages in particular, and immune cells in general, has advanced, it is becoming increasingly clear that this is a highly dynamic process. Attempts to classify macrophages in subpopulations have proven intricate, as they seem to be both a continuum and a heterogeneous mix of subpopulations that do not always coincide with the M1/M2 paradigm (Sica and Mantovani, 2012; Wang et al., 2013; Mendoza-Coronel and Ortega, 2017; Grosjean et al., 2021; Munoz-Rojas et al., 2021). The analysis of our regulatory network suggests that there are independent circuits composed of receptors, transcription factors, and cytokines that activate in response to the signals in the environment. Some of these circuits inhibit each other (IFN-γ and IL-10), others are mostly independent (VEGF and IL-10), and others have more complex relationships (IL-6). Furthermore, these circuits can have dynamically stable oscillations, which affect not only the production of downstream cytokines, but also the crosstalk with other pathways. We propose that, on the one hand, when the circuits inhibit each other, we can expect a clear separation in the expression levels of the molecules involved (IL-12 and IL-10) and almost no plasticity, which creates pseudo-populations for those specific markers. On the other hand, when the circuits are independent or modulate each other in context-specific ways, the result is a continuum of expression for those markers, as seen in the M2-like family of attractors. In this case, given that the circuits are mostly independent, we should expect a higher level of “plasticity” as the circuits are activated or inhibited depending on the environmental signals. These circuits are further modulated by the microenvironment, the initial state of the cell, and stochastic effects. Focusing on the active regulatory circuits could give us a framework to comprehend the biological functions of macrophages in specific conditions while considering the environment, heterogeneity, and plasticity of these cells. This could have a profound impact on our understanding of the pathogenic mechanisms in certain diseases. For example, in patients with Crohn’s disease there is a clear difference between macrophage populations from the intestinal mucosa and from the mesenteric fat tissue. In the former, TLR-4, IL-1b and IL-6 protein levels are higher compared to those in patients with non-inflammatory disease; while in the latter there is no such increase. The authors of the study attribute these differences to the micro-environment, which in the case of intestinal macrophages is largely determined by the interaction with the microbiota. As a consequence, there is an anomalous up-regulation of the signaling pathways that result in the production of inflammatory mediators. Hence, a network like the one we devised could be of great value to understand this type of heterogeneous scenarios, helping improve medical care towards the design of treatments with side effects noticeably reduced in comparison to the ones currently prescribed.

The model also allowed us to determine the key nodes of the network. When subjected to knock-out or over-expression experiments, IL12_out, IL10_out, STAT1, STAT6, and IL-10 mediated STAT3 activation (STAT3*) had the most notable effect, especially IL-12 and IL-10, as they are cell type markers. Also, *in vivo* cells are subjected to transient changes in extrinsic cytokine levels or stochastic effects in signaling pathways and transcriptional regulation, which we simulated as transient perturbations. The nodes that caused more transitions between cell types were: STAT1, STAT3*, STAT6, IC_e, FCGR, and SOCS1. On the other hand, IL12_out, VEGF_out, NECA_e, IL1R, and TLR4 had the least effect. STAT1, STAT6, and STAT3* activation has a higher number of out-going edges and directly or indirectly modulates the activation and inhibition of different network circuits, which explains their key roles within the network dynamics.

While the Boolean nature of the model favors the study of how the structure of the regulatory network determines cellular behavior, it also limits the scope of the analysis. The model uses discrete values for the nodes, severely restricting our understanding of how the range of expression levels observed in macrophages is generated. Furthermore, the model uses discrete time steps and synchronous actualization for very different processes like signaling, which can take minutes, and transcription, which can take hours. Most cyclic attractors of size two or three were unstable, but we did recover asynchronous cyclic attractors of size six or bigger, however understanding their biological implications is still an open question. The model is also deterministic, and the perturbation analysis, while sufficient to determine the possible transitions, is not a true stochastic analysis. This is particularly important as the internal state of the cell and random noise probably have an important role in the emergence of heterogeneous populations. Further models using differential equations or stochastic methods are warranted.

The model also oversimplifies the NF-kβ pathway to a degree where the predicted and experimental mutants are not in accordance. Additionally, our network would benefit from the inclusion of multiple molecules like TNF-ɑ, TGF-β, TLRs, NODs, and MyD88. In fact, it will be necessary to incorporate these molecules to integrate the model to other cell types and create integrative immune models. Finally, the number of environments used was limited to reported polarizing conditions and mixed environments (Sica and Mantovani, 2012; Wang et al., 2013; Mendoza-Coronel and Ortega, 2017; Grosjean et al., 2021; Munoz-Rojas et al., 2021). Thus, it will be interesting to see if the model can be extended to study diseases with complex immune profiles, like cancer, *tuberculosis*, or COVID-19.

## Data Availability

The datasets presented in this study can be found in online repositories. The names of the repository/repositories and accession number(s) can be found below: https://github.com/mar-esther23/Macrophage_Differentiation.

## References

[B1] Albert R. Thakar J. (2014). Boolean Modeling: A Logic-Based Dynamic Approach for Understanding Signaling and Regulatory Networks and for Making Useful Predictions. Wires Syst. Biol. Med. 6, 353–369. 10.1002/wsbm.1273 25269159

[B2] Alvarez-Buylla Roces M. E. Martınez-Garcıa J. C. Davila-Velderrain J. Domınguez-Huttinger E. Martınez-Sanchez M. E. (2018). Medical Systems Biology. Adv. Exp. Med. Biol. 1069, 1. 10.1007/978-3-319-89354-9_1 30076565

[B3] Anders C. B. Lawton T. M. W. Smith H. L. Garret J. Doucette M. M. Ammons M. C. B. (2021). Use of Integrated Metabolomics, Transcriptomics, and Signal Protein Profile to Characterize the Effector Function and Associated Metabotype of Polarized Macrophage Phenotypes. J. Leukoc. Bio 111, 667–693. 10.1002/JLB.6A1120-744R 34374126 PMC8825884

[B4] Arora S. Dev K. Agarwal B. Das P. Syed M. A. (2018). Macrophages: Their Role, Activation and Polarization in Pulmonary Diseases. Immunobiology 223, 383–396. 10.1016/J.IMBIO.2017.11.001 29146235 PMC7114886

[B5] At W. Hl J. Pj C. Am W. (2011). Tight Control of STAT5 Activity Determines Human CD34-Derived Interstitial Dendritic Cell and Langerhans Cell Development. J. Immunol. 186, 7016–7024. 10.4049/JIMMUNOL.1003977 21602494

[B6] Avila-Ponce de León U. Vázquez-Jiménez A. Matadamas-Guzman M. Pelayo R. Resendis-Antonio O. (2021). Transcriptional and Microenvironmental Landscape of Macrophage Transition in Cancer: A Boolean Analysis. Front. Immunol. 12, 2110. 10.3389/fimmu.2021.642842 PMC8222808 34177892

[B7] Bally A. P. R. Lu P. Tang Y. Austin J. W. Scharer C. D. Ahmed R. (2015). NF-κB Regulates PD-1 Expression in Macrophages. J.I. 194, 4545–4554. 10.4049/jimmunol.1402550 PMC4402259 25810391

[B8] Bouhlel M. A. Derudas B. Rigamonti E. Dièvart R. Brozek J. Haulon S. (2007). PPARγ Activation Primes Human Monocytes into Alternative M2 Macrophages with Anti-inflammatory Properties. Cell Metab. 6, 137–143. 10.1016/j.cmet.2007.06.010 17681149

[B9] Chang Q. Bournazou E. Sansone P. Berishaj M. Gao S. P. Daly L. (2013). The IL-6/JAK/Stat3 Feed-Forward Loop Drives Tumorigenesis and Metastasis. Neoplasia 15, 848–IN45. 10.1593/NEO.13706 23814496 PMC3689247

[B10] Chawla A. (2010). Control of Macrophage Activation and Function by PPARs. Circ. Res. 106, 1559–1569. 10.1161/CIRCRESAHA.110.216523 20508200 PMC2897247

[B11] Cheng Q. J. Ohta S. Sheu K. M. Spreafico R. Adelaja A. Taylor B. (2021). NF-κB Dynamics Determine the Stimulus Specificity of Epigenomic Reprogramming in Macrophages. Science 372 (6548), 1349–1353. 10.1126/science.abc0269 34140389 PMC8489855

[B12] Colin S. Chinetti-Gbaguidi G. Staels B. (2014). Macrophage Phenotypes in Atherosclerosis. Immunol. Rev. 262, 153–166. 10.1111/imr.12218 25319333

[B13] Derrida B. Pomeau Y. (1986). Random Networks of Automata: a Simple Annealed Approximation. Europhys. Lett. 1 (2), 45–49. 10.1209/0295-5075/1/2/001

[B14] Foey A. D. (2014). Macrophages - Masters of Immune Activation, Suppression and Deviation. Immune Response Activation, 276 Chapter 5. https://www.intechopen.com/books/3811 . 10.5772/57541

[B15] Funes S. C. Rios M. Escobar-Vera J. Kalergis A. M. (2018). Implications of Macrophage Polarization in Autoimmunity. Immunology 154, 186–195. 10.1111/IMM.12910 29455468 PMC5980179

[B16] Gong M. Zhuo X. Ma A. (2017). STAT6 Upregulation Promotes M2 Macrophage Polarization to Suppress Atherosclerosis. Med. Sci. Monit. Basic Res. 23, 240–249. 10.12659/msmbr.904014 28615615 PMC5484610

[B17] Gonzalez a. G. Naldi A. Sánchez L. Thieffry D. Chaouiya C. (2006). GINsim: a Software Suite for the Qualitative Modelling, Simulation and Analysis of Regulatory Networks. Biosystems 84, 91–100. 10.1016/j.biosystems.2005.10.003 16434137

[B18] Gordon S. Martinez F. O. (2010). Alternative Activation of Macrophages: Mechanism and Functions. Immunity 32, 593–604. 10.1016/j.immuni.2010.05.007 20510870

[B19] Grinberg S. Hasko G. Wu D. Leibovich S. J. (2009). Suppression of PLCβ2 by Endotoxin Plays a Role in the Adenosine A2A Receptor-Mediated Switch of Macrophages from an Inflammatory to an Angiogenic Phenotype. Am. J. Pathol. 175, 2439–2453. 10.2353/ajpath.2009.090290 19850892 PMC2789640

[B20] Grosjean A. Venteclef N. Dalmas E. (2021). Understanding the Heterogeneity and Functions of Metabolic Tissue Macrophages. Semin. Cell Developmental Biol. 119, 130–139. 10.1016/j.semcdb.2021.09.002 34561168

[B21] Hamilton J. A. (2019). GM-CSF-Dependent Inflammatory Pathways. Front. Immunol. 10. 10.3389/fimmu.2019.02055 PMC6737278 31552022

[B22] Huang X. Li Y. Fu M. Xin H.-B. (2018). Polarizing Macrophages *In Vitro* . Methods Mol. Biol. 1784, 119–126. 10.1007/978-1-4939-7837-3_12 29761394 PMC8875934

[B23] Hutchins A. P. Diez D. Miranda-Saavedra D. (2013). The IL-10/STAT3-mediated Anti-inflammatory Response: Recent Developments and Future Challenges. Brief. Funct. Genomics 12, 489–498. 10.1093/bfgp/elt028 23943603 PMC3838198

[B24] Kauffman S. A. (1969). Metabolic Stability and Epigenesis in Randomly Constructed Genetic Nets. J. Theor. Biol. 22, 437–467. 10.1016/0022-5193(69)90015-0 5803332

[B25] Kim H. S. Kim D. C. Kim H. M. Kwon H. J. Kwon S. J. Kang S. J. (2015). STAT1 Deficiency Redirects IFN Signalling toward Suppression of TLR Response through a Feedback Activation of STAT3. Sci. Rep. 5 (1), 13414–13415. 10.1038/srep13414 26299368 PMC4547106

[B26] Kumar V. (2019). Macrophages: The Potent Immunoregulatory Innate Immune Cells. Macrophage Activation - Biol. Dis. 10.5772/INTECHOPEN.88013

[B27] Lawrence T. Natoli G. (2011). Transcriptional Regulation of Macrophage Polarization: Enabling Diversity with Identity. Nat. Rev. Immunol. 11, 750–761. 10.1038/nri3088 22025054

[B29] Lehtonen A. Matikainen S. Miettinen M. Julkunen I. (2002). Granulocyte-macrophage colony-stimulating Factor (GM-CSF)-induced STAT5 Activation and Target-Gene Expression during Human Monocyte/macrophage Differentiation. J. Leukoc. Biol. 71, 511–519. 10.1189/jlb.71.3.511 11867689

[B30] Leibovich S. J. Chen J.-F. Pinhal-Enfield G. Belem P. C. Elson G. Rosania A. (2002). Synergistic Up-Regulation of Vascular Endothelial Growth Factor Expression in Murine Macrophages by Adenosine A2A Receptor Agonists and Endotoxin. Am. J. Pathol. 160, 2231–2244. 10.1016/S0002-9440(10)61170-4 12057925 PMC1850844

[B31] Li X. Jolly M. K. George J. T. Pienta K. J. Levine H. (2019). Computational Modeling of the Crosstalk between Macrophage Polarization and Tumor Cell Plasticity in the Tumor Microenvironment. Front. Oncol. 9, 10. 10.3389/fonc.2019.00010 30729096 PMC6351454

[B32] Liquitaya-Montiel A. J. Mendoza L. (2018). Dynamical Analysis of the Regulatory Network Controlling Natural Killer Cells Differentiation. Front. Physiol. 9, 1029. 10.3389/fphys.2018.01029 30116200 PMC6082967

[B33] Liu L. Zhao Y. Xie K. Sun X. Jiang L. Gao Y. (2014). Estrogen Inhibits LPS-Induced IL-6 Production in Macrophages Partially via the Nongenomic Pathway. Immunological Invest. 43, 693–704. 10.3109/08820139.2014.917095 24960169

[B34] Locati M. Curtale G. Mantovani A. (2020). Diversity, Mechanisms, and Significance of Macrophage Plasticity. Annu. Rev. Pathol. Mech. Dis. 15, 123–147. 10.1146/annurev-pathmechdis-012418-012718 PMC7176483 31530089

[B35] Lucas M. Zhang X. Prasanna V. Mosser D. M. (2005). ERK Activation Following Macrophage FcγR Ligation Leads to Chromatin Modifications at the IL-10 Locus. J. Immunol. 175, 469–477. 10.4049/jimmunol.175.1.469 15972681

[B36] Luo Y. Pollard J. W. Casadevall A. (2010). Fcγ Receptor Cross-Linking Stimulates Cell Proliferation of Macrophages via the ERK Pathway. J. Biol. Chem. 285, 4232–4242. 10.1074/jbc.M109.037168 19996316 PMC2823562

[B37] Ma X. Yan W. Zheng H. Du Q. Zhang L. Ban Y. (2015). Regulation of IL-10 and IL-12 Production and Function in Macrophages and Dendritic Cells. F1000Res 4, 1465 41465. 10.12688/f1000research.7010.1 PMC4754024 26918147

[B38] Majai G. Sarang Z. Csomós K. Zahuczky G. Fésüs L. (2007). Pparγ-dependent Regulation of Human Macrophages in Phagocytosis of Apoptotic Cells. Eur. J. Immunol. 37 (5), 1343–1354. 10.1002/eji.200636398 17407194

[B39] Martinez-Sanchez M. E. Enciso J. Arias Del Angel J. (2018). BoolNetPerturb: Perturb Boolean Networks. R package version 0.2. GitHub. Available at: https://github.com/mar-esther23/boolnet-perturb.git

[B40] Martinez-Sanchez M. E. Mendoza L. Villarreal C. Alvarez-Buylla E. R. (2015). A Minimal Regulatory Network of Extrinsic and Intrinsic Factors Recovers Observed Patterns of CD4+ T Cell Differentiation and Plasticity. Plos Comput. Biol. 11, e1004324. 10.1371/journal.pcbi.1004324 26090929 PMC4475012

[B41] Matsuyama T. Kubli S. P. Yoshinaga S. K. Pfeffer K. Mak T. W. (2020). An Aberrant STAT Pathway Is central to COVID-19. Cell Death Differ 27, 3209–3225. 10.1038/s41418-020-00633-7 33037393 PMC7545020

[B42] Mendoza-Coronel E. Ortega E. (2017). Macrophage Polarization Modulates FcγR- and CD13-Mediated Phagocytosis and Reactive Oxygen Species Production, Independently of Receptor Membrane Expression. Front. Immunol. 8, 303. 10.3389/fimmu.2017.00303 28396660 PMC5366847

[B43] Merad M. Martin J. C. (2020). Pathological Inflammation in Patients with COVID-19: a Key Role for Monocytes and Macrophages. Nat. Rev. Immunol. 20, 355–362. 10.1038/s41577-020-0331-4 32376901 PMC7201395

[B44] Muñoz-Rojas A. R. Kelsey I. Pappalardo J. L. Chen M. Miller-Jensen K. (2021). Co-stimulation with Opposing Macrophage Polarization Cues Leads to Orthogonal Secretion Programs in Individual Cells. Nat. Commun. 12, 301. 10.1038/s41467-020-20540-2 33436596 PMC7804107

[B45] Müssel C. Hopfensitz M. Kestler H. A. (2010). BoolNet-an R Package for Generation, Reconstruction and Analysis of Boolean Networks. Bioinformatics 26, 1378–1380. 10.1093/bioinformatics/btq124 20378558

[B46] Naldi A. Carneiro J. Chaouiya C. Thieffry D. (2010). Diversity and Plasticity of Th Cell Types Predicted from Regulatory Network Modelling. Plos Comput. Biol. 6, e1000912. 10.1371/journal.pcbi.1000912 20824124 PMC2932677

[B47] Palma A. Jarrah A. S. Tieri P. Cesareni G. Castiglione F. (2018). Gene Regulatory Network Modeling of Macrophage Differentiation Corroborates the Continuum Hypothesis of Polarization States. Front. Physiol. 9, 1659. 10.3389/fphys.2018.01659 30546316 PMC6278720

[B48] Park B. S. Song D. H. Kim H. M. Choi B.-S. Lee H. Lee J.-O. (2009). The Structural Basis of Lipopolysaccharide Recognition by the TLR4-MD-2 Complex. Nature 458, 1191–1195. 10.1038/nature07830 19252480

[B49] Petrina M. Martin J. Basta S. (2021). Granulocyte Macrophage colony-stimulating Factor Has Come of Age: From a Vaccine Adjuvant to Antiviral Immunotherapy. Cytokine Growth Factor. Rev. 59, 101–110. 10.1016/j.cytogfr.2021.01.001 33593661 PMC8064670

[B50] Ramírez C. Mendoza L. (2018). Phenotypic Stability and Plasticity in GMP-Derived Cells as Determined by Their Underlying Regulatory Network. Bioinformatics 34, 1174–1182. 10.1093/bioinformatics/btx736 29186334

[B51] Ramirez R. Herrera A. M. Ramirez J. Qian C. Melton D. W. Shireman P. K. (2019). Deriving a Boolean Dynamics to Reveal Macrophage Activation with *In Vitro* Temporal Cytokine Expression Profiles. BMC Bioinformatics 20, 725. 10.1186/s12859-019-3304-5 31852428 PMC6921543

[B52] Saez-Rodriguez J. Simeoni L. Lindquist J. A. Hemenway R. Bommhardt U. Arndt B. (2007). A Logical Model Provides Insights into T Cell Receptor Signaling. Plos Comput. Biol. 3, e163–1590. 10.1371/journal.pcbi.0030163 17722974 PMC1950951

[B53] Sica A. Mantovani A. (2012). Macrophage Plasticity and Polarization: *In Vivo* Veritas. J. Clin. Invest. 122, 787–795. 10.1172/JCI59643 22378047 PMC3287223

[B54] Tarique A. A. Logan J. Thomas E. Holt P. G. Sly P. D. Fantino E. (2015). Phenotypic, Functional, and Plasticity Features of Classical and Alternatively Activated Human Macrophages. Am. J. Respir. Cell Mol Biol 53 (5), 676–688. 10.1165/rcmb.2015-0012oc 25870903

[B55] Vannella K. M. Wynn T. A. (2017). Mechanisms of Organ Injury and Repair by Macrophages. Annu. Rev. Physiol. 79, 593–617. 10.1146/annurev-physiol-022516-034356 27959618

[B56] Varol C. Mildner A. Jung S. (2015). Macrophages: Development and Tissue Specialization. Annu. Rev. Immunol.Annual Reviews) 33, 643–675. 10.1146/annurev-immunol-032414-112220 25861979

[B57] Wang L. x. Zhang S. x. Wu H. j. Rong X. l. Guo J. (2019). M2b Macrophage Polarization and its Roles in Diseases. J. Leukoc. Biol. 106, 345–358. 10.1002/JLB.3RU1018-378RR 30576000 PMC7379745

[B58] Wang Y. Van Boxel-Dezaire A. H. H. Cheon H. Yang J. Stark G. R. (2013). STAT3 Activation in Response to IL-6 Is Prolonged by the Binding of IL-6 Receptor to EGF Receptor. Proc. Natl. Acad. Sci. U.S.A. 110, 16975–16980. 10.1073/pnas.1315862110 24082147 PMC3801081

[B59] Weber A. Wasiliew P. Kracht M. (2010). Interleukin-1 (IL-1) Pathway. Sci. Signal. 3, cm1. 10.1126/scisignal.3105cm1 20086235

[B60] Wilson H. M. (2014). SOCS Proteins in Macrophage Polarization and Function. Front. Immunol. 5, 357. 10.3389/fimmu.2014.00357 25120543 PMC4112788

[B61] Yoshimura A. Naka T. Kubo M. (2007). SOCS Proteins, Cytokine Signalling and Immune Regulation. Nat. Rev. Immunol. 7, 454–465. 10.1038/nri2093 17525754

[B62] Zhang Y. Liu S. Liu J. Zhang T. Shen Q. Yu Y. (2009). Immune Complex/Ig Negatively Regulate TLR4-Triggered Inflammatory Response in Macrophages through FcγRIIb-dependent PGE2 Production. J. Immunol. 182, 554–562. 10.4049/jimmunol.182.1.554 19109188

