# Supplementary figures and images for: Cyclic Attractors Are Critical for Macrophage Differentiation, Heterogeneity, and Plasticity
Source: Front Mol Biosci. 2022 Apr 11;9:807228. doi: 10.3389/fmolb.2022.807228 (PMC9035596; doi:10.3389/fmolb.2022.807228)

# Attractors of size 1

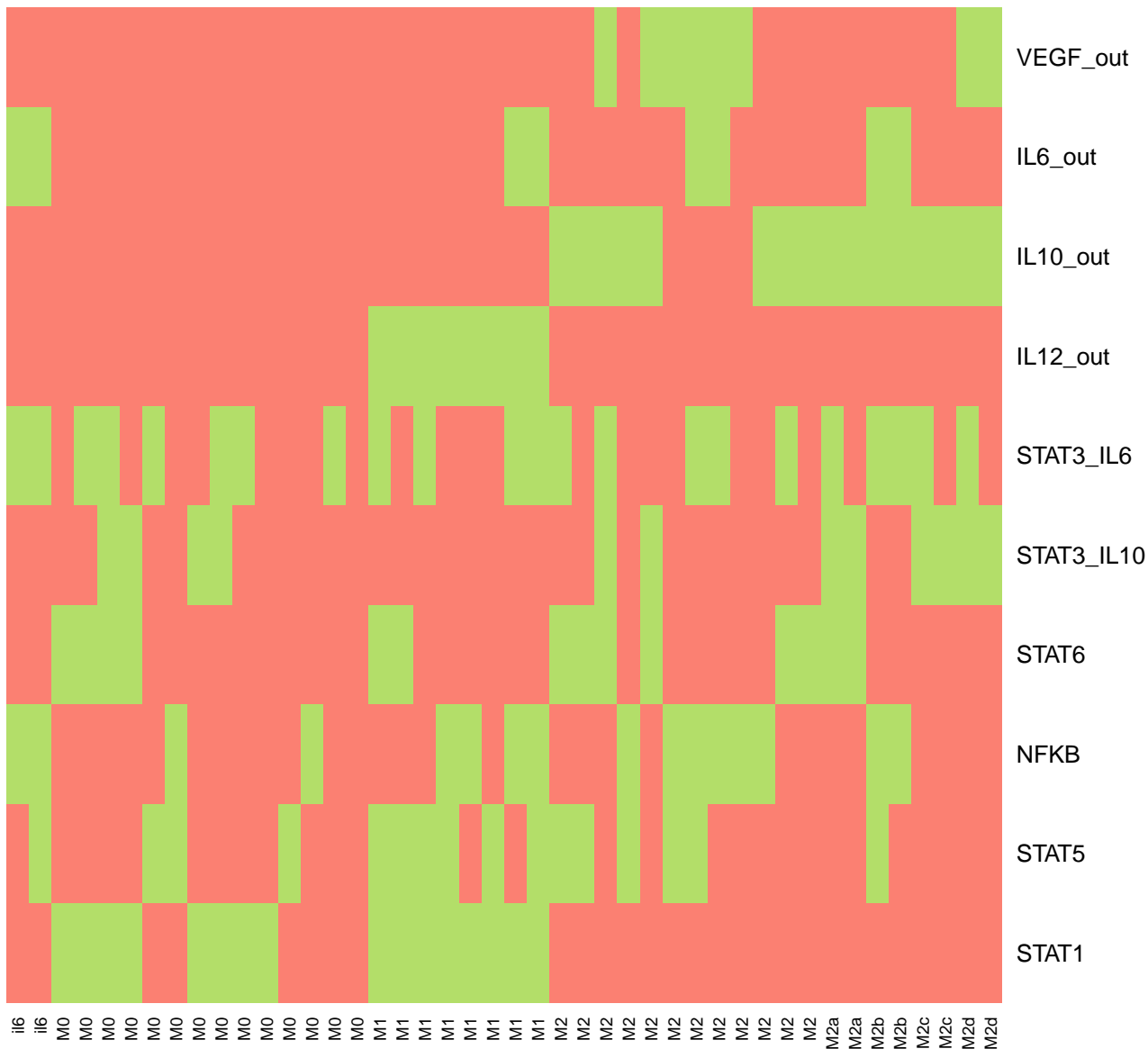

# Attractors of size 2

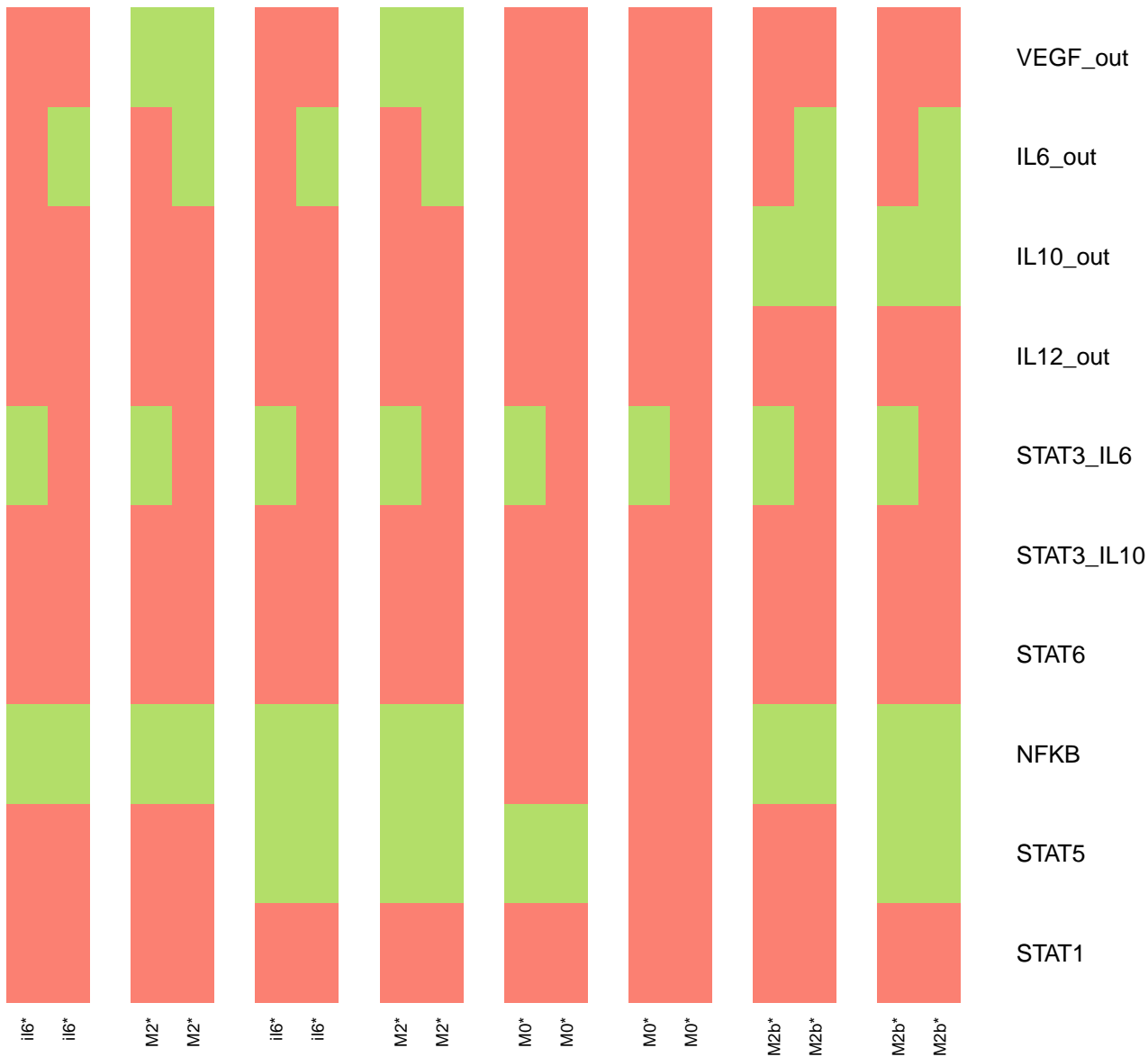

# Attractors of size 3

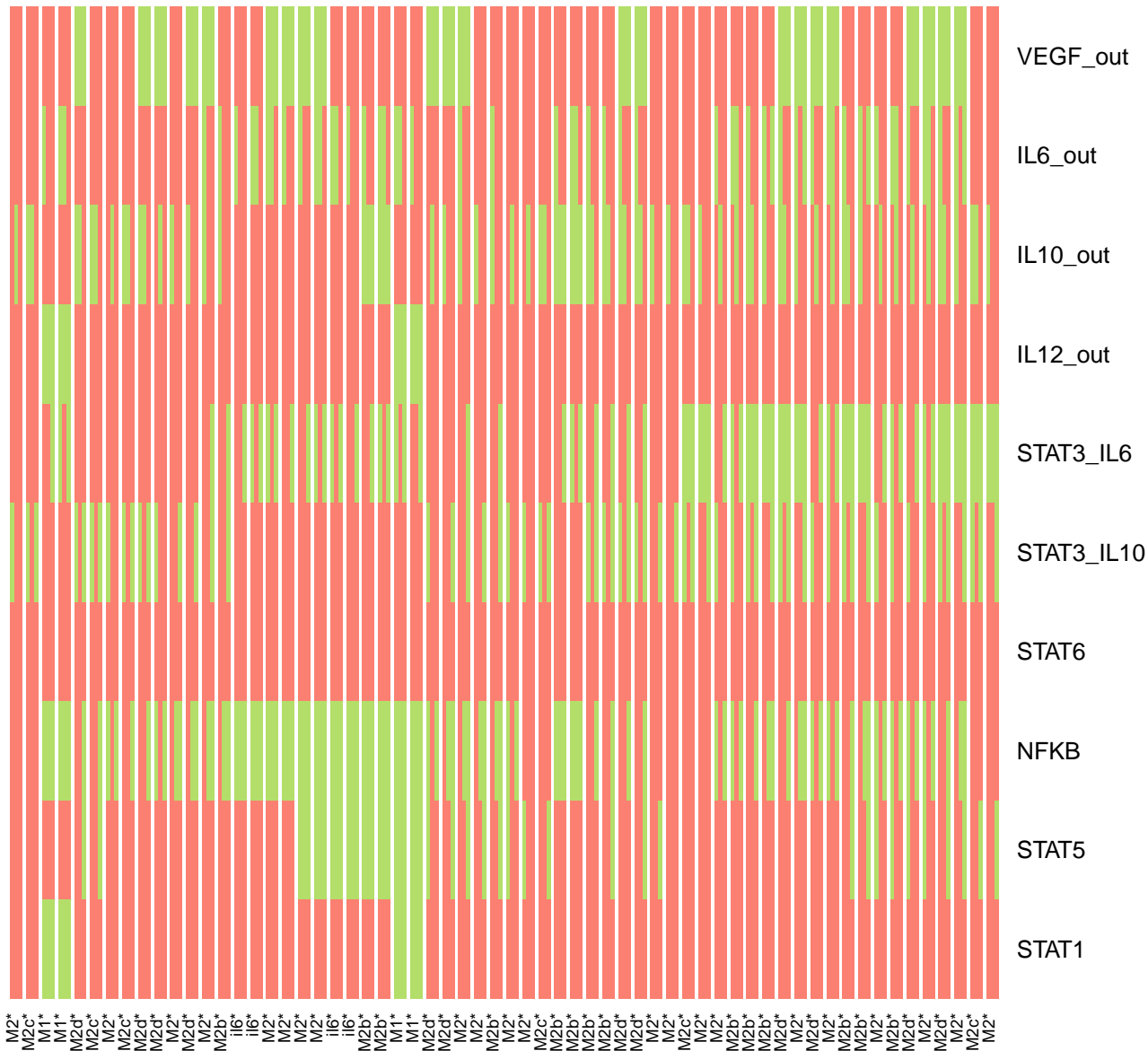

# Attractors of size 6

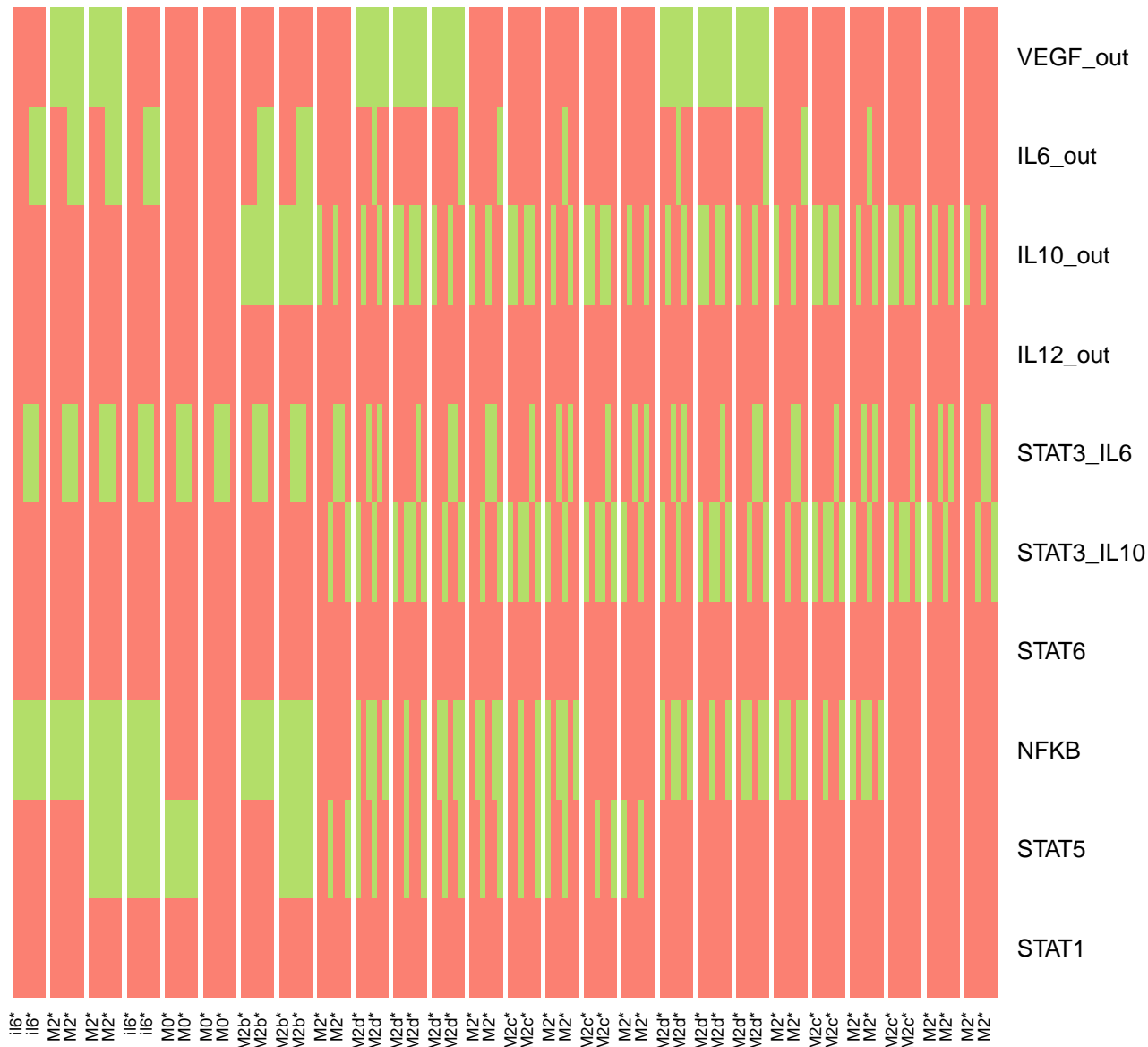

Supplement: Supplementary file 3 [file Presentation1.PDF]

# Macrophage mutants

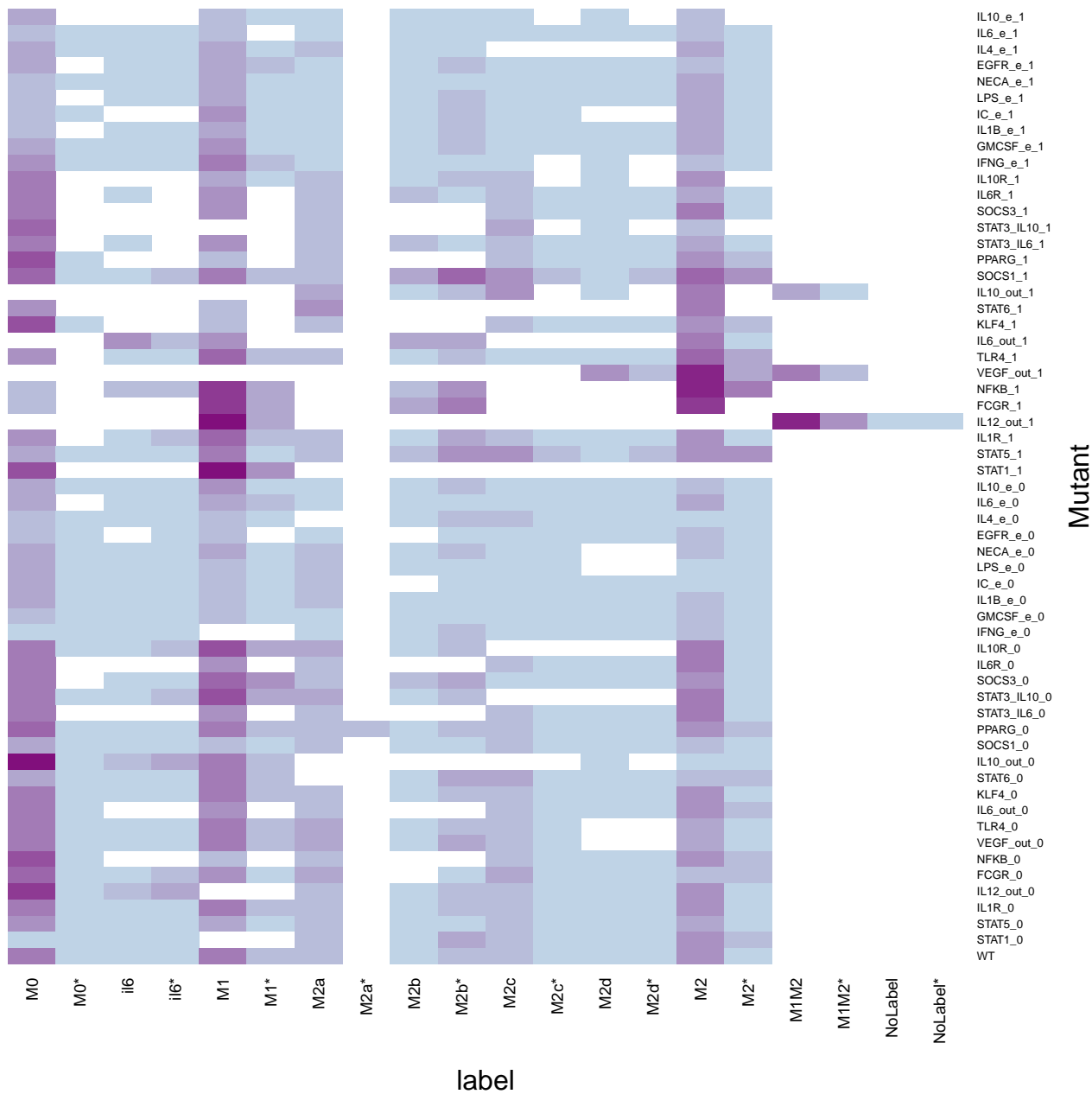

Supplement: Supplementary file 9 [file Presentation2.PDF]

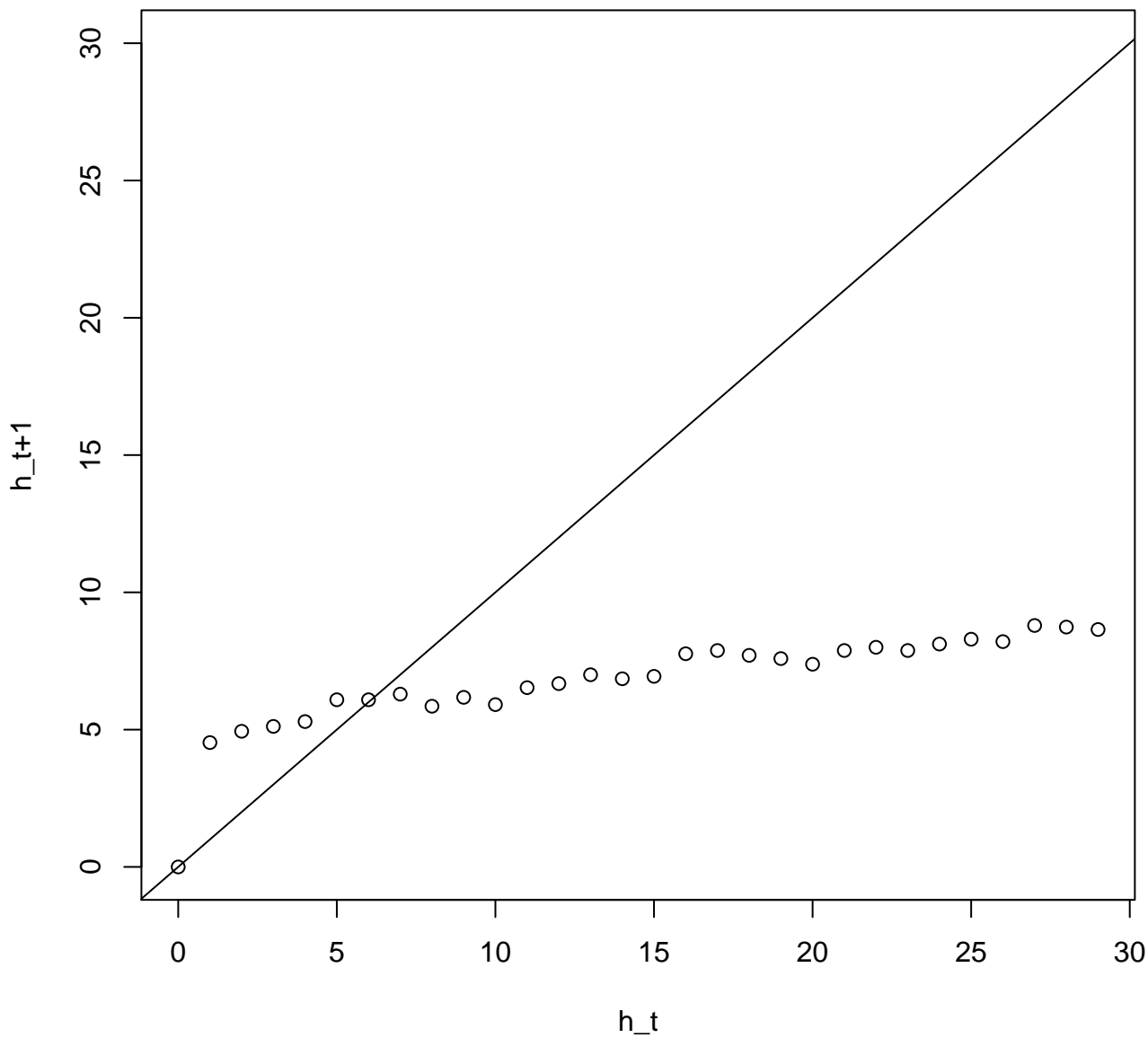

Supplement: Supplementary file 12 [file Presentation3.PDF]
